# Combination Therapy With CGF and Microneedling‐Assisted Compound Betamethasone for Resistant Alopecia Areata: A Pilot Study

**DOI:** 10.1111/jocd.16591

**Published:** 2024-09-21

**Authors:** Lingling Jia, Changjiang Zhao, Hongyi Zhang, Hua Jiang, Jiachao Xiong, Yufei Li

**Affiliations:** ^1^ Department of Plastic Surgery, Shanghai East Hospital Tongji University School of Medicine Shanghai China

**Keywords:** alopecia, concentrated growth factor, microneedling, resistant alopecia areata

## Abstract

**Background:**

Alopecia areata (AA), an autoimmune disorder characterized by hair loss, can be particularly difficult to manage when patients do not respond to standard therapeutic approaches such as topical or injectable corticosteroids, contact immunotherapy, and systemic treatments. In instances where these conventional therapies prove ineffective, alternative or adjunctive treatments are sought. Concentrated growth factor (CGF) and microneedling (MN)‐assisted drug delivery are promising methods for the treatment of different dermatological diseases.

**Objective:**

This study aimed to assess the practical benefits and the safety aspects of utilizing a dual treatment approach involving CGF and MN‐assisted compound betamethasone for patients suffering from resistant AA that are unresponsive to conventional medical interventions.

**Material and Methods:**

This retrospective study was based on evaluations of seven patients with refractory AA treated with CGF and MN‐assisted compound betamethasone from July 2021 to December 2023. The efficacy of treatment was assessed by extents of hair regrowth percentages of involved areas.

**Results:**

Among the seven enrolled patients with refractory AA, a notable outcome was observed where one patient (14.3%) achieved a regrowth of hair by over 50%, while six patients (85.7%) exhibited complete recovery without any systemic or local adverse effects. Furthermore, the difference in SALT scores between baseline, and the final visit for all patients was found to be statistically significant, substantiating the therapeutic efficacy of the intervention employed.

**Conclusion:**

The present study demonstrated that the synergistic application of CGF in conjunction with MN‐assisted compound betamethasone may constitute a promising and well‐tolerated therapeutic modality for refractory AA, offering a potentially efficacious and safe treatment alternative.

## 1 Introduction

Alopecia areata (AA) represents a multifaceted autoimmune condition characterized by non‐scarring alopecia. The disease typically presents as an abrupt onset of asymmetric, circumscribed hair loss patches on either the scalp or other body regions, culminating in smooth, sharply demarcated bald areas devoid of epidermal atrophy. AA is traditionally categorized into patchy alopecia, alopecia totalis, and alopecia universalis. However, a more comprehensive classification should take into account the disease duration and, with regard to patchy alopecia areata, the extent of the hair loss [1]. And the classification as refractory is reserved for cases that exhibited an inadequate response to at least two distinct therapeutic modalities [2], that is, in cases where a patient fails to demonstrate a therapeutic response within the 3–6 month interval, the possibility of treatment resistance should be entertained [3]. Currently, the pathogenesis of AA, especially refractory AA, is not fully understood. It may be related to the collapse of hair follicle immune privilege collapse, auto‐active cytotoxic CD8 T cells, and interferon‐γ‐driven immune response [3, 4]. All available treatments for refractory AA are palliative in nature, including intralesional steroids, systemic corticosteroids, contact immunotherapy, photochemotherapy, and JAK inhibitors [1]. These treatments aim to control ongoing episodes of hair loss but do not offer a cure. Despite extensive research efforts, resistant AA continues to pose a significant challenge due to the inherent limitations of current therapies, with their efficacies being highly variable and largely dependent on the severity and duration of the condition. Furthermore, the frequent occurrence of relapse following treatment cessation underscores a pressing need for the development of safer and more efficacious therapeutic strategies in managing resistant AA. In recent times, novel treatment modalities have gained attention, with concentrated growth factor (CGF) and microneedling (MN) emerging as promising alternatives.

Depending on the specific centrifugation parameters, platelet concentrates are classified into platelet‐rich plasma (PRP), platelet‐rich fibrin (PRF), and CGF. Autologous CGF, initially developed by Sacco [5], represents the latest generation of platelet products that contains a high concentration of growth factors and CD34‐positive stem cells. Microneedling is a minimally invasive intervention, typically executed using either an electronic pen apparatus or a mechanical micro‐roller instrument, which incorporates ultrafine needles with skin puncture depths ranging from 0.5 to 2.5 mm. This procedure enables controlled and targeted stimulation of the skin's physiological processes without extensive tissue damage [6]. To date, CGF and MN have demonstrated synergistic potential when combined with other hair growth stimulants, such as minoxidil and platelet‐rich plasma, effectively promoting hair regrowth. While the application of these therapies in the treatment of alopecia has been extensively researched in the context of androgenetic alopecia (AGA), their investigation in resistant AA remains relatively underexplored [7, 8].

## 2 Materials and Methods

**FIGURE 1:**
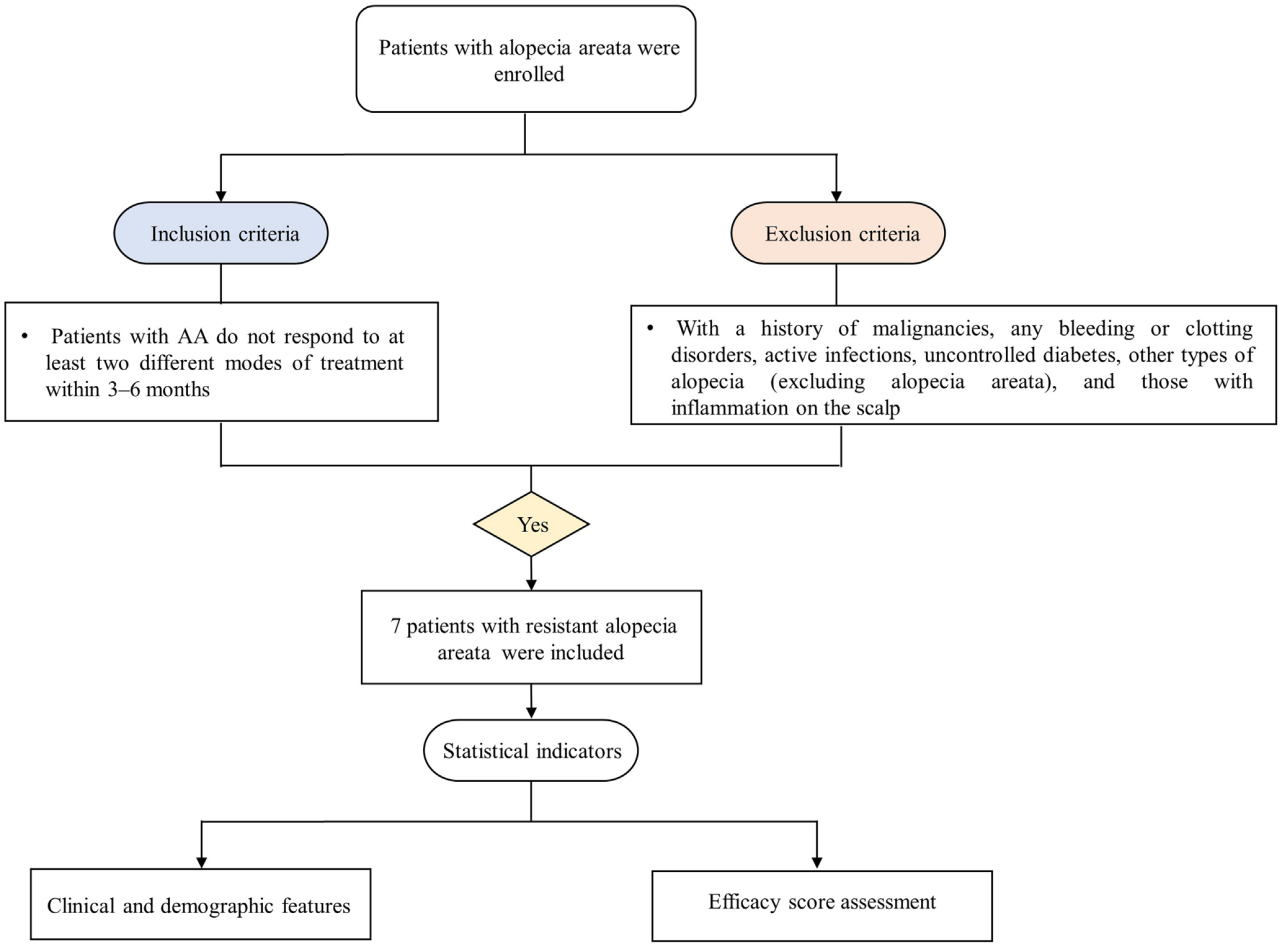
Flow diagram. The chart shows patients’ inclusion and exclusion in the study.

The data collection for this study was conducted within the timeframe extending from July 2021 to December 2023. The current investigation enrolled a cohort of seven adult patients (aged 18 years and above) with refractory AA (resistant to at least two distinct therapeutic modalities for more than 6 months) affecting the scalp. All participants had discontinued the use of intralesional corticosteroid injections, topical creams, and minoxidil lotion at least 2 months prior to their enrollment in the study. Exclusion criteria were rigorously implemented to ensure the exclusion of patients with specific health conditions from the study population. These criteria encompassed individuals with a history of malignancies, bleeding or clotting disorders, ongoing infectious processes, uncontrolled diabetes mellitus, other forms of alopecia beyond AA, and those presenting with active inflammation on the scalp (Figure 1 illustrates the patient selection process). All participants underwent meticulous general medical examinations to exclude the presence of any concurrent autoimmune or systemic pathologies, while comprehensive dermatological assessments were performed to ascertain the precise type and distribution of AA lesions. All enrolled patients provided written informed consent for their participation in this study, as well as consented to the utilization of photographic documentation to track their therapeutic progress.

## 3 Treatment Protocol

**FIGURE 2:**
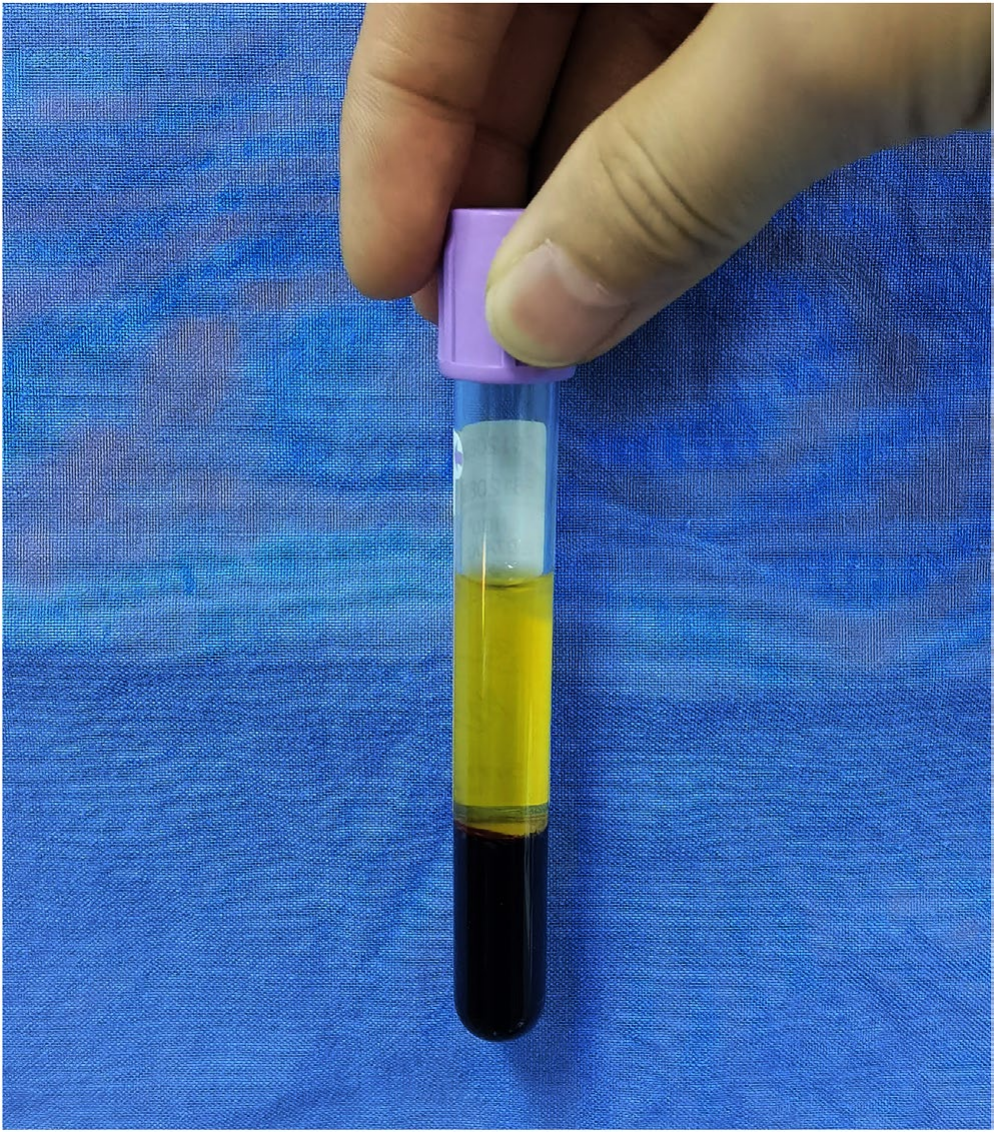
CGF. Three blood fractions were obtained after centrifugation with anticoagulant: (1) the upper layer represented by PPP; (2) the middle layer or buffy coat represented by CGF; and (3) the lower red blood cell layer.

Concentrated growth factor was prepared using differential centrifugation. Briefly, 18 mL of fresh blood was collected in vacutainer tubes containing EDTA‐K2 as an anticoagulant (K.S MEDICAL; Zhejiang, China) under aseptic conditions. The collected blood sample underwent centrifugation following a specific protocol [9]: 30 s acceleration, 2 min at 2700 rpm, 4 min at 2400 rpm, 4 min at 2700 rpm, 3 min at 3000 rpm, and 36 s deceleration and stop. This centrifugation process resulted in the blood separating into three layers (Figure 2). The upper platelet poor plasma (PPP) and trapped red blood cells were discarded, while the middle layer or buffy coat, totaling 4–6 mL, was collected as CGF.

Prior to the therapeutic intervention, the target scalp areas were subjected to meticulous aseptic preparation using normal saline and iodophor solution, and then anesthetized by local anesthetic injections in a circular fashion. Injections of CGF, at a dosage of 0.1–0.2 mL/cm^2^, were then administered into the alopecia areas of the scalp at a depth of 2 mm. After the injections are over with, MN was done using dermaroller device having 540 needles of 1.5 mm length which was rolled over the region diagonally, vertically, and horizontally in a controlled manner, repeating the procedure four to five times along each axis to ensure comprehensive coverage. This micro‐invasive procedure resulted in pinpoint bleeding, which was considered an endpoint indicative of the intended dermal stimulation. Thereafter, compounded betamethasone was applied topically to the corresponding scalp region, followed by gentle massage with fingertips, which would facilitate compounded betamethasone getting absorbed from the micro‐punctures developed post MN. The main component of compounded betamethasone used in the study is betamethasone dipropionate. Each 1 mL of compounded betamethasone contains 5 mg of betamethasone dipropionate and 2 mg of betamethasone sodium phosphate. The average treatment sessions for all patients were 7.7 (range from 6 to 15) at 1‐or 2‐week intervals to ensure adequate therapeutic exposure.

## 4 Outcome Assessment

Dermoscopic evaluations, alongside photographic documentation, were conducted at three critical timepoints: baseline (prior to the initiation of treatment), during the therapeutic course, and a minimum of 3 months following the conclusion of treatment. At each visit, dermoscopy (JieDa, China) was used to appraise treatment outcomes according to established criteria. The presence of yellow dots, broken hairs, black dots, and tapering hairs served as indicators of ongoing disease activity, while the emergence of upright regrowing hair and terminal hair signified improvement in the patient's condition. Disease severity and treatment efficacy were systematically evaluated utilizing the Severity of Alopecia Tool (SALT) scoring system. The clinical response was operationally defined as a > 50% increase in hair regrowth relative to baseline measurements.

## 5 Adverse Events

All adverse events encountered during the study period, encompassing manifestations such as pruritus, persistent pain, erythema, swelling, papules, and scarring, were meticulously documented at each scheduled patient visit.

### 5.1 Statistical Analysis

Statistical analyses were executed using GraphPad Prism 8.0 software (GraphPad Software Co., San Diego, CA, USA), where the differences between variables were evaluated employing the nonparametric Mann–Whitney *U* test. The level of statistical significance was set at a two‐tailed *p*‐value of less than 0.05.

## 6 Results

### 6.1 Subject Demographics

In the current study, we meticulously assessed a cohort of seven patients diagnosed with refractory AA (four males and three females). The mean age of the study participants was 36 years, with an age distribution that extended from a minimum of 23 years to a maximum of 52 years. The mean SALT score at the commencement of the combined treatment regimen was 11.0. All participants in this study had previous treatments including triamcinolone local injection (seven patients, 100%), topical corticosteroid (seven patients, 100%), narrow‐band ultraviolet phototherapy (five patients, 71.4%), topical minoxidil (four patients, 57.1%), methotrexate (two patients, 28.6%), and systemic steroid (one patient, 14.3%). Of particular significance is the absence of any concurrent dermatological disorders reported by the patients. The data are summarized in Table 1.

**TABLE 1:** Demographic characteristics of the seven patients with refractory AA.

Characteristics	Total patients (*n* = 7)
Mean age, years	36 (23–52)
Sex	
Male Female	4 (57.1%) 3 (42.9%)
Family history	0 (0.0)
Concomitant autoimmune diseases	0 (0.0)
Mean treatment sessions	7.7 (6–15)
Mean SALT score at baseline of study Mean SALT score after follow‐up	11.0% 2.0%
Previous treatments	
Triamcinolone local injection Topical corticosteroid Narrow‐band ultraviolet phototherapy Topical minoxidil Methotrexate Systemic steroid	7 (100%) 7 (100%) 5 (71.4%) 4 (57.1%) 2 (28.6%) 1 (14.3%)

### 6.2 Efficacies of Treatments With CGF Plus MN

Among the seven patients enrolled in this study, one participant (14.3%) exhibited a regrowth of hair exceeding 50%, and six subjects (85.7%) manifested substantial therapeutic benefits, as evidenced by the complete resolution of their refractory AA. Significantly, the statistical analysis demonstrated a marked difference in SALT scores between baseline and the final visit for patients (*p* < 0.05). The duration of follow‐up subsequent to the cessation of treatment varied between 3 and 9 months, with an average follow‐up period of 5.6 months. Upon meticulous dermoscopic assessment, a discernible difference was detected in the pretreatment and posttreatment clinical findings. Notably, following the treatment intervention, there was a marked diminution in the prevalence of characteristic yellow dots, black dots, cone‐shaped hairs, and empty hair follicles, which typically denote active AA. Concurrently, a statistically significant augmentation was observed in both the number of upright regrowing hair shafts and terminal hairs, thereby indicating a favorable therapeutic response and the restoration of normal hair growth cycles.

**FIGURE 3:**
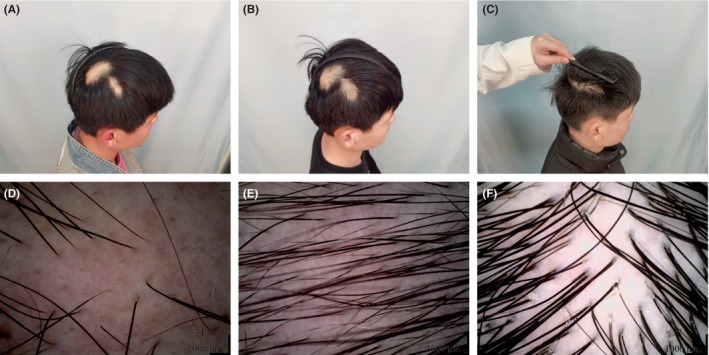
A 23‐year‐old male showing refractory AA. (A) Baseline. (B) After eight sessions. (C) Follow‐up for 9 months after the last session. (D) Dermoscopic pictures at baseline: Yellow dots, black dots, and cone‐shaped hairs. (E, F) Dermoscopic pictures after eight sessions and 9 months: Absence of signs indicative of AA activity, and increase in hair density.

**FIGURE 4:**
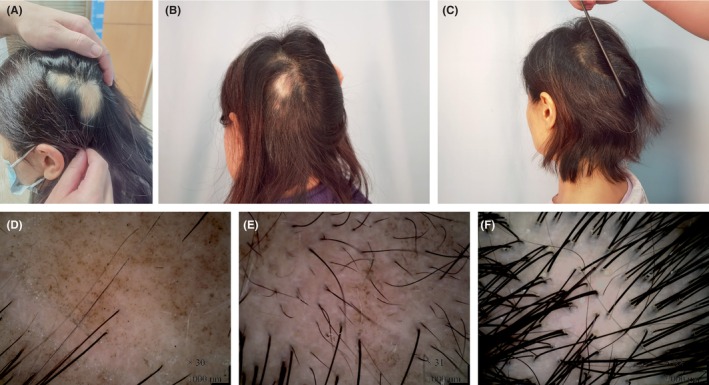
A 52‐year‐old female showing refractory AA. (A) Baseline. (B) After 15 sessions. (C) Follow‐up for 5 months after the last session. (D) Dermoscopic picture at baseline: Yellow dots, black dots, and cone‐shaped hairs, broken hairs. (E, F) Dermoscopic pictures after 15 sessions and 5 months: Absence of signs indicative of AA activity, and increase in hair density.

Systematic clinical and dermoscopic photography was conducted for two representative patients (Figures 3 and 4).

### 6.3 Assessment of Adverse Events

Throughout the entire follow‐up period, no clinically significant adverse events were either reported or detected among the subjects, encompassing but not limited to pruritus, protracted pain, erythema, swelling, papules, or scarring. Moreover, no occurrences of atrophy or telangiectasia emerged as delayed‐onset complications in any of the subjects.

## 7 Discussion

In the conventional armamentarium of medical therapies for AA, a spectrum of modalities has been employed, encompassing topical and intralesional corticosteroids as frontline treatments, alongside systemic corticosteroids and other immunosuppressive agents. Contact immunotherapy, ultraviolet B narrowband phototherapy, and light‐based interventions have also been integral components in the therapeutic landscape [10]. Intralesional corticosteroid injections constitute the primary therapeutic modality for patients with AA. This intervention protocol typically spans a period of 6 weeks to 6 months, entailing regular administrations of corticosteroids at sessions conducted every 3–4 weeks, employing a concentration gradient that customarily falls within the spectrum of 2.5–10 mg/mL per injection episode. However, this treatment method may be not effective for resistant AA and may lead to adverse reactions such as skin atrophy [11]. The current challenge is to determine and develop an optimal strategy that can achieve the therapeutic effect of refractory AA compared to current treatment models, while minimizing the occurrence of adverse reactions.

Concentrated growth factor is referred to as the “third‐generation” PRP due to its enhanced properties and potential benefits in regenerative medicine. The centrifugation method used to prepare CGF leads to a product with a higher concentration of growth factors and a more organized, softer, and thinner fibrin matrix compared to traditional PRP, which is more conducive to tissue regeneration and repair [12]. Steward et al. [13] reported that combining PRP with MN and CGF gel could enhance microcirculation and stimulate hair growth in 20 patients. Tan et al.'s [14] findings indicate that injecting CGF into the scalp combining with topical minoxidil resulted in significantly increased hair density and growth rate compared to using minoxidil alone, suggesting a potentially synergistic effect when CGF is used in conjunction with conventional treatments. Similarly, Zhao et al.'s [15] research supports these findings as they also discovered that CGF treatment significantly improves the condition of hair loss and increases hair diameter in individuals suffering from AGA. In our own study, we administered CGF injections directly into the areas affected by alopecia. The rationale behind this approach lies in CGF's ability to continuously and slowly release growth factors, thus preventing a rapid, short‐lived “burst” effect. This slow‐release mechanism is thought to be beneficial because it could potentially extend the period during which the therapeutic biological activity is maintained, thereby promoting more effective and sustained hair regrowth. In addition, the rationale for incorporating MN was to enhance the penetration and targeted delivery of betamethasone into the dermis, the primary site of active inflammation in resistant AA, thereby ensuring efficacious engagement with the affected tissue. In contrast, conventional intralesional corticosteroid injections may exhibit a propensity to infiltrate the subcutaneous adipose tissue rather than optimally delivering the medication to its intended target within the dermis. This could potentially result in a reduced concentration of the therapeutic agent reaching the site of active inflammation in resistant AA. An ancillary advantage of MN is its capacity to stimulate collagen synthesis, a biological response that may serve to mitigate the dermal atrophy commonly associated with corticosteroid use [16]. Of the seven enrolled patients with refractory AA, a notably encouraging outcome was observed in nine cases (85.7%), all of whom achieved complete hair regrowth without encountering any serious adverse events. However, one patient (14.3%) with a lesion duration of 2 years and an extended history of irregular treatment, potentially leading to follicular necrosis, experienced partial recovery with ≥ 50% hair regrowth. Consequently, this particular case did not achieve complete therapeutic success. Over the course of a 5.6‐month follow‐up period, a remarkable 85.7% (*n* = 6) of patients demonstrated complete remission. Although the exact mechanisms underlying how CGF works in tandem with MN‐assisted compound betamethasone to stimulate hair growth have yet to be fully understood, there are several plausible hypotheses based on existing scientific data: first, CGF, rich in CD34‐positive stem cells and various growth factors, provides a reservoir of biological molecules that can be gradually released into the scalp tissues. This continuous supply likely fosters an optimal environment for hair follicle regeneration and sustains hair growth over a longer period. Second, MN creates a micro‐wound environment that can trigger a cascade of events leading to the upregulation of hair growth‐related genes, such as Vascular Endothelial Growth Factor (VEGF), β‐catenin, Wnt3a, and Wnt10b [17]. These genes play pivotal roles in hair cycle regulation, hair shaft production, and hair follicle development. Third, compound betamethasone, when delivered through MN‐assisted transdermal pathways, experiences augmented permeation and absorption into the skin. This not only ensures a deeper and more efficient delivery to the targeted hair follicular units but also potentially increases the drug's bioavailability at the site of action, thereby amplifying its therapeutic effect on hair growth stimulation [18, 19].

Dermoscopy, while traditionally recognized for its diagnostic utility in AA, also holds considerable promise as a noninvasive tool for the comprehensive monitoring and longitudinal evaluation of therapeutic responses in patients with AA [20]. In the present study, at the initiation of treatment, the most frequently observed dermoscopic features included yellow dots, characteristic tapering hairs (notably, exclamation mark hairs and cone‐shaped hairs), black dots, and absent follicular openings. These manifestations served as hallmark dermoscopic signs indicative of AA activity in the study population. Upon completion of the treatment regimen, patches treated with CGF in conjunction with MN‐assisted compound betamethasone exhibited a significant diminution of the previously observed dermoscopic manifestations. The final dermoscopy assessment conducted during the 5.6‐month follow‐up period revealed that all characteristic features indicative of AA were no longer present in our patients. These dermoscopic changes effectively corroborated the clinical response observed in patients subjected to the combined therapy. Furthermore, these findings suggest that such alterations in dermoscopic signs can serve as favorable prognostic indicators for AA treatment outcomes.

As regards the side effects, no significant adverse events were encountered among the study cohort. Mild discomfort and erythema were transiently experienced by a minority of patients, which aligns with the findings reported by Zhao et al. [15] and Hou et al. [21].

## 8 Conclusion

The utilization of CGF in conjunction with MN‐assisted compound betamethasone presents a promising avenue for improving outcomes in resistant AA patients who have shown limited responsiveness to conventional therapies. However, it is imperative to acknowledge that the present study is constrained by its relatively small sample size and the brevity of the follow‐up period. Future research endeavors should encompass large‐scale, multicenter, double‐blind, randomized controlled trials designed to thoroughly evaluate the long‐term therapeutic benefits and safety profile of the combined CGF and MN for resistant AA patients. The present findings and insights are poised to expedite the broader application of CGF and MN technology in alopecia disorders, thereby potentially offering substantial benefits to a large patient population suffering from resistant AA.

## Author Contributions

Y.L., J.X., and H.J. designed the overall study; L.J., C.Z., and H.Z. performed the experiment and drafted the initial version of the manuscript; Y.L. revised manuscript, and all authors approved the final version of the manuscript.

## Ethics Statement

The study has been carried out in accordance with the Code of Ethics of the World Medical Association for experiments involving humans.

## Consent

Written informed consents and photograph consents were obtained from all patients for scientific publication.

## Conflicts of Interest

The authors declare no conflicts of interest.

## Data Availability

The data that support the findings of this study are available from the corresponding author upon reasonable request.
